# Localization of PD‐L1 on single cancer cells by iSERS microscopy with Au/Au core/satellite nanoparticles

**DOI:** 10.1002/jbio.201960034

**Published:** 2020-01-01

**Authors:** Elzbieta Stepula, Matthias König, Xin‐Ping Wang, Janina Levermann, Tobias Schimming, Sabine Kasimir‐Bauer, Bastian Schilling, Sebastian Schlücker

**Affiliations:** ^1^ Department of Chemistry and Center for Nanointegration Duisburg‐Essen (CENIDE) University of Duisburg‐Essen Essen Germany; ^2^ Department of Gynecology and Obstetrics, University Hospital Essen University of Duisburg‐Essen Essen Germany; ^3^ Department of Dermatology, University Hospital University of Duisburg‐Essen Essen Germany; ^4^ Department of Dermatology University Hospital in Würzburg Würzburg Germany

**Keywords:** gold nanoparticles, PD‐L1, Raman, SERS

## Abstract

Programmed cell death‐ligand 1 (PD‐L1) is an important predictive biomarker. The detection of PD‐L1 can be crucial for patients with advanced cancer where the use of immunotherapy is considered. Here, we demonstrate the use of immuno‐SERS microscopy (iSERS) for localizing PD‐L1 on single cancer SkBr‐3 cells. A central advantage of iSERS is that the disturbing autofluorescence from cells and tissues can be efficiently minimized by red to near‐infrared laser excitation. In this study we employed Au/Au core/satellite nanoparticles as SERS nanotags because of their remarkable signal brightness and colloidal stability upon red laser excitation. False‐color iSERS images of the positive and negative controls clearly reveal the specific localization of PD‐L1 with SERS nanotag‐labeled antibodies.

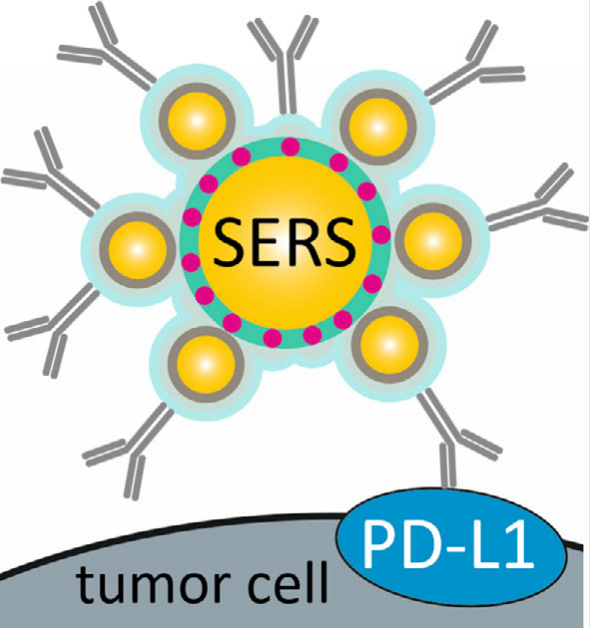

## Graphical abstract

iSERS (SERS=surface‐enhanced Raman scattering) microscopy is an emerging Raman‐based staining technique for the selective localization of target proteins on cells and tissues using antibody‐SERS nanotag conjugates. In this contribution we demonstrate the feasibility of iSERS for imaging of programmed cell deathligand 1 (PD‐L1), an important predictive biomarker, on single SkBr‐3 breast cancer cells.

## 1 INTRODUCTION

Programmed cell death‐ligand 1 (PD‐L1) plays a crucial role in immune regulation and is expressed in numerous healthy tissues, for example, in the placenta 1. PD‐L1 also mediates immune evasion in many solid and hematological malignancies including malignant melanoma and breast cancer 2, 3, 4. Blockade of programmed cell death‐1 (PD‐1) and its ligand PD‐L1 is used as an immunotherapy employing so called immune checkpoint inhibitors (ICI). ICI has been shown to prolong progression‐free (PFS) and overall survival (OS) in different cancers and has become a common denominator in modern oncology 5, 6, 7. This impressive and wide clinical activity can be accompanied by severe immune‐related adverse events (irAE) and although rare, treatment‐related deaths 8, 9. In addition, maximum benefit might be restricted to patients with certain characteristics 10 and detection of biomarkers including but not limited to T cell infiltration, mutational load and PD‐L1 expression 11, 12, 13. Very recently, a prospective, randomized phase 3 trial in advanced untreated triple‐negative breast cancer (TNBC) showed increased PFS in patients receiving the PD‐L1 blocking antibody atezolizumab plus nab‐paclitaxel in comparison to nab‐paclitaxel plus placebo 14. Increased OS by adding atezolizumab to nab‐paclitaxel was only reported for PD‐L1 positive tumors. Methods and assays for detecting biomarkers are therefore key to use ICI with maximum clinical benefit.

Nowadays immunohistochemistry (IHC) remains the gold standard for localizing PD‐L1 on cancerous tissue. However, the evaluation of IHC slides from, for example, skin cancer patients is very difficult even for experienced pathologists for reasons listed below 11. Melanoma develops from melanocytes cells, with a color which is similar to that of the product of the HRP enzyme reaction. Immunofluorescent staining is not really an attractive alternative to IHC due to the very strong autofluorescence of the sample caused by the brown pigment 15.

A promising alternative to IHC and IF is immuno‐SERS microscopy (iSERS) 16. This emerging technique is based on surface‐enhanced Raman scattering (SERS) for optical readout and circumnavigates the problems associated with IHC and IF 17, 18. In iSERS antigens are localized via target recognition by antibodies as in other immuno‐based methods. However, instead of labeling the antibody with an enzyme or a fluorophore, a molecularly functionalized noble metallic nanoparticle is employed 19. In our work we used Au/Au core/satellite particles with Raman‐active reporter molecules on the surface 20. The unique Raman signature of the molecule can be enhanced by the gold nanostructure upon resonant optical excitation and is used for the identification of the particles 21. The SERS nanotag‐antibody conjugates are then localized on single cells or tissue specimen by Raman microspectroscopy. With the help of iSERS it is possible to overcome the limitations of the standard techniques for localizing PD‐L1 on skin tissue since the use of SERS nanotags instead of HRP enzyme or fluorophores has several advantages. As mentioned before, specimens with malignant melanoma exhibit strong autofluorescence. This can be overcome by the application of red to near‐infrared laser excitation which minimizes the disturbing effect of autofluorescence and improves the image contrast 22, 23. In contrast to fluorescent dyes, the nanotags are chemically stable and inert against photobleaching. Overall, iSERS is a fast and reproducible method for protein localization on single cancer cells 24, 25.

## 2 MATERIALS AND METHODS/EXPERIMENTAL

### 2.1 Materials

Tetrachloroauric acid (HAuCl_4_ ∙ 3 H_2_O), 1‐ethyl‐3‐(3‐dimethylaminopropyl)carbodiimide (EDC), *N*‐hydroxysulfosuccinimide sodium salt (sulfo‐NHS), bovine serum albumin (BSA), (11‐mercaptoundecyl)‐*N*,*N*,*N*‐trimethylammonium bromide (MUTAB), poly(sodium‐4‐styrenesulfonate) (PSS), 7‐mercapto‐4‐methylcoumarin (MMC) and 4‐(2‐hydroxyethyl)‐1‐piperazine ethane sulfonic acid (HEPES) were purchased from Sigma Aldrich. Phosphate buffered saline (PBS) was prepared by dissolving powder purchased from Biochrom GmbH, Germany, in Milli‐Q water. Paraformaldehyde (PFA) solution (4% in PBS) was obtained from Affymetrix/Thermo Fisher, Germany. Rabbit anti‐PD‐L1 antibody [clone 28‐8] was obtained from Abcam, Germany. Goat anti‐rabbit antibody was purchased from Thermo Fisher, Germany. Ultrapure water (18.2 MΩ cm, Millipore) was used throughout the experiments.

### 2.2 Cell culture and preparation

The human breast cancer cell line SkBr–3 was purchased from American Type Culture Collection (ATCC, Manassas, Virginia). SkBr–3 cells were cultured in McCoy's 5a Medium (Gibco by Life Technologies, Thermo Fisher Scientifc, Waltham, Massachusetts) supplemented with 10% (vol/vol) fetal calf serum (Gibco), 1% (vol/vol) penicillin‐streptomycin (Sigma Aldrich, St. Louis, Missouri), 7.5% (vol/vol) sodium bicarbonate (Gibco) and 200 mM L‐glutamine (Gibco). Cells were grown at 37°C in a humidified atmosphere with 5% CO_2_. Subcultivation was performed with trypsin‐EDTA solution (0.05% trypsin, 0.02% EDTA, Sigma Aldrich). A total number of ca. 10^5^ SkBr3 cells were cytospinned on glass slides coated with poly‐l‐lysine (R. Langenbrinck, Emmendingen, Germany) at 1000 g for 6 minutes and air‐dried overnight at RT.

### 2.3 Synthesis of SERS nanotags and antibody conjugates

The Au/Au core/satellite nanoparticles comprise a positively charged 50 nm gold nanosphere (core) 26, 27 with a smooth surface (AuNSs), coated with the linker molecule MUTAB and the Raman reporter MMC, as well as negatively charged 30 nm gold nanoparticles (satellites) (AuNP) 28. The assemblies are stabilized by a PEG shell (HS‐PEG‐COOH) to avoid non–specific binding of the SERS nanotags to cells. The last step is the bioconjugation of antibodies to the SERS nanotags. The carboxyl groups were activated by EDC/sulfo‐NHS for 25 minutes at RT. The rabbit anti‐PD‐L1 antibody was added to the activated SERS colloid and incubated for 2 hours at RT and then overnight at 4°C. The suspension was washed with 2% BSA/PBS four times in order to remove any unreacted antibodies and then suspended in 2% BSA/PBS solution for subsequent cell staining.

### 2.4 Cell fixation, blocking and staining

The cytospinned cell slides were washed with PBS buffer three times to remove the remaining culture medium. Next, 4% PFA was added onto the cell slides and incubated for 15 minutes at RT for fixation. After incubation, the cell slides were washed with PBS buffer three times to remove an excess of 4% PFA. In order to unmask the antigen, heat‐induced epitope retrieval was performed. The cell slides were heated at 95°C for 10 minutes in citrate buffer pH 6, cooled down and washed in PBS buffer. Then 2% BSA/PBS solution was added to the cells slides as a blocking reagent for 2 hours at RT to reduce any unspecific binding and background. The primary antibody was incubated on the SkBr–3 cell slides for 1 hour at 37°C. Afterward, the slides were stained with the SERS‐labeled secondary antibody for 8 hours under shaking. Finally, the cell slides were washed three times with PBS buffer and once with water to remove any unbound SERS nanotags and then mounted.

For the IF staining the primary antibody was incubated on the SkBr–3 cell slides for 1 hour at 37°C. Then, the fluorescent‐labeled (AF647 dye) secondary goat anti‐rabbit antibody was incubated for 40 minutes at RT. Finally, the cell slides were washed three times with PBS buffer and once with water before mounting.

### 2.5 iSERS microscopy on single breast cancer cells

Single cells were identified in the bright field for imaging of selected areas by confocal Raman microscopy (WITec alpha 300 R, grating monochromator with *f* = 30 cm focal length, EM‐CCD grating). A 40× air objective with cover glass correction and a NA of 0.6 (Olympus LUC Plan FLN) was used. The 632.8 nm radiation from a He‐Ne laser with a power of 1.2 mW at the sample was employed for exciting Raman scattering. The integration time per pixel was 100 ms. For the localization of PD‐L1 on single cells, the cells were at first identified in the bright‐field. Next an area was selected for the SERS mapping and investigated with an integration time of 100 ms and a laser power of 1.2 mW at the sample. After performing the mapping experiments the recorded SERS spectra were processed. Raw spectra were smoothed via the Savitzky‐Golay algorithm (7th order polynomial, 31 points). Then a baseline correction (Whittaker‐Henderson procedure 29) was performed. Mean spectra with error bars were calculated using a MATLAB algorithm (shadedErrorBars). For each pixel the maximum intensity of the Raman peak centered at ca. 1590 cm^−1^ was determined by using a Lorentzian line profile and a nonlinear least square algorithm in order to generate the corresponding SERS false‐color images.

## 3 RESULTS AND DISCUSSION

### 3.1 Characterization of Au/Au core/satellite particles (SERS nanotags)

**Figure 1:**
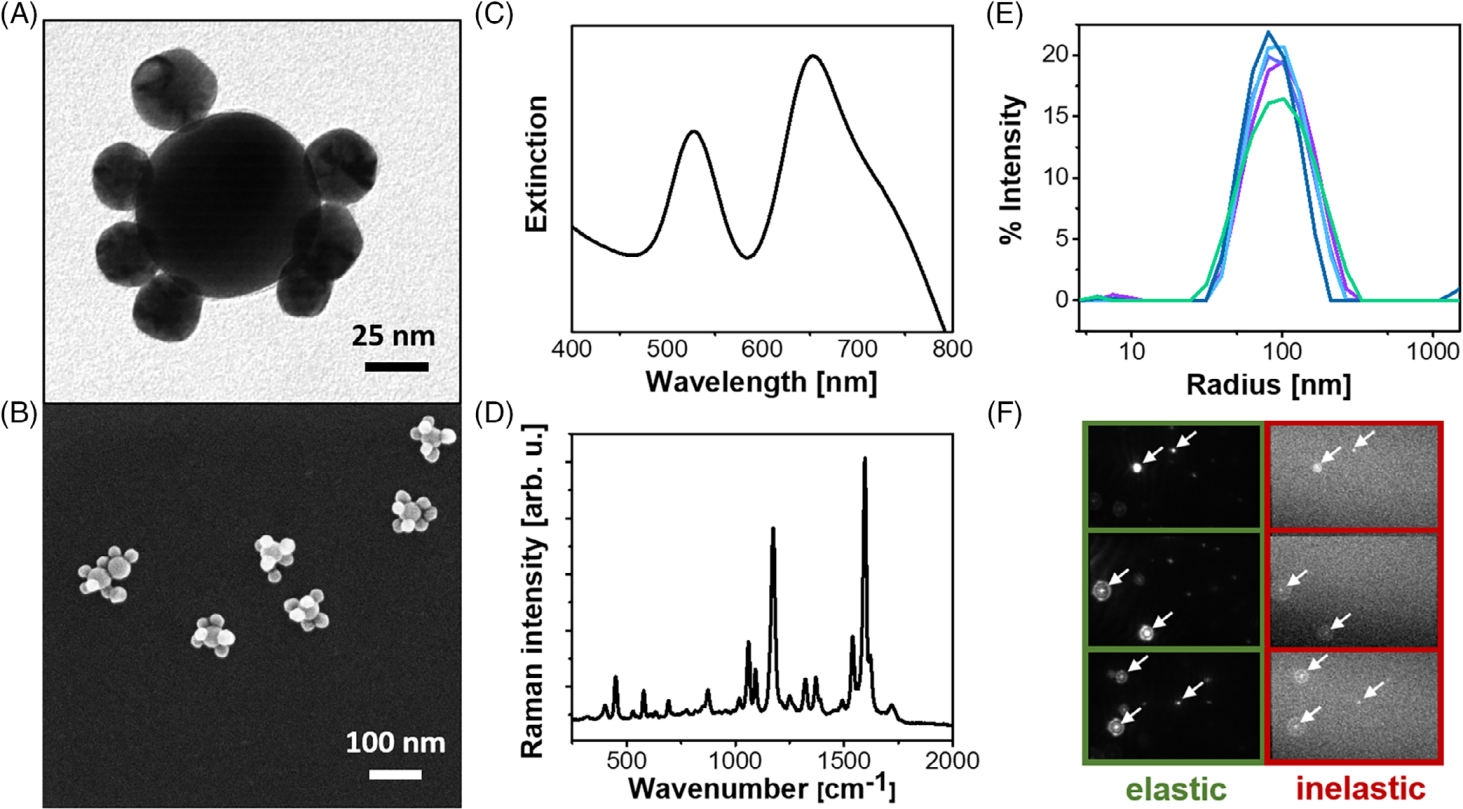
Characterization of Au/Au core/satellite particles. A, TEM image; B, SEM image; C, extinction spectrum; D, SERS spectrum of MMC; E, size distribution; F, correlative single‐particle real‐time imaging. MMC, 7‐mercapto‐4‐methylcoumarin; SEM, scanning electron microscopy; SERS, surface‐enhanced Raman scattering; TEM, transmission electron microscopy

Figure 1A shows a transmission electron microscopy (TEM) image of a single Au/Au core/satellite particle and Figure 1B a scanning electron microscopy (SEM) image. The UV/Vis extinction spectrum of Au/Au core/satellite particles exhibits two plasmon peaks. The peak at ca. 700 nm is assigned to a plasmon coupling mode of the core and the satellites 30, 31. A SERS spectrum with the characteristic Raman peak of MMC at 1590 and 1200 cm^−1^ is shown in Figure 1D. Based on DFT calculations, we assign these two normal modes to a C═C stretching mode and a ring breathing mode, both with dominant contributions from the phenyl ring with the thiol group. The hydrodynamic radius of the particles before the bioconjugation, as determined by dynamic light scattering, is (88 ± 2) nm. Correlative Rayleigh/Raman single‐particle real‐time imaging of the Au/Au core/satellite particles using a home‐built optical setup was performed for demonstrating their single‐particle brightness 32. Figure 1F shows snapshots from a video recorded during the measurement. Individual particles can be observed in both the elastic (left) and the inelastic (right) channel which confirms the SERS activity of Au/Au core/satellite particles at the single‐particle level 28, 33.

### 3.2 Specificity of anti‐PD‐L1‐antibody/SERS nanotag‐conjugates in single cell iSERS microscopy

The most important aspect in immunostaining is the unambiguous demonstration of binding specificity. This is especially crucial in iSERS microscopy since the relatively large SERS nanotags (~100 nm compared to ca. 15 nm for an IgG) may affect the binding affinity of the corresponding antibody.

**Figure 2:**
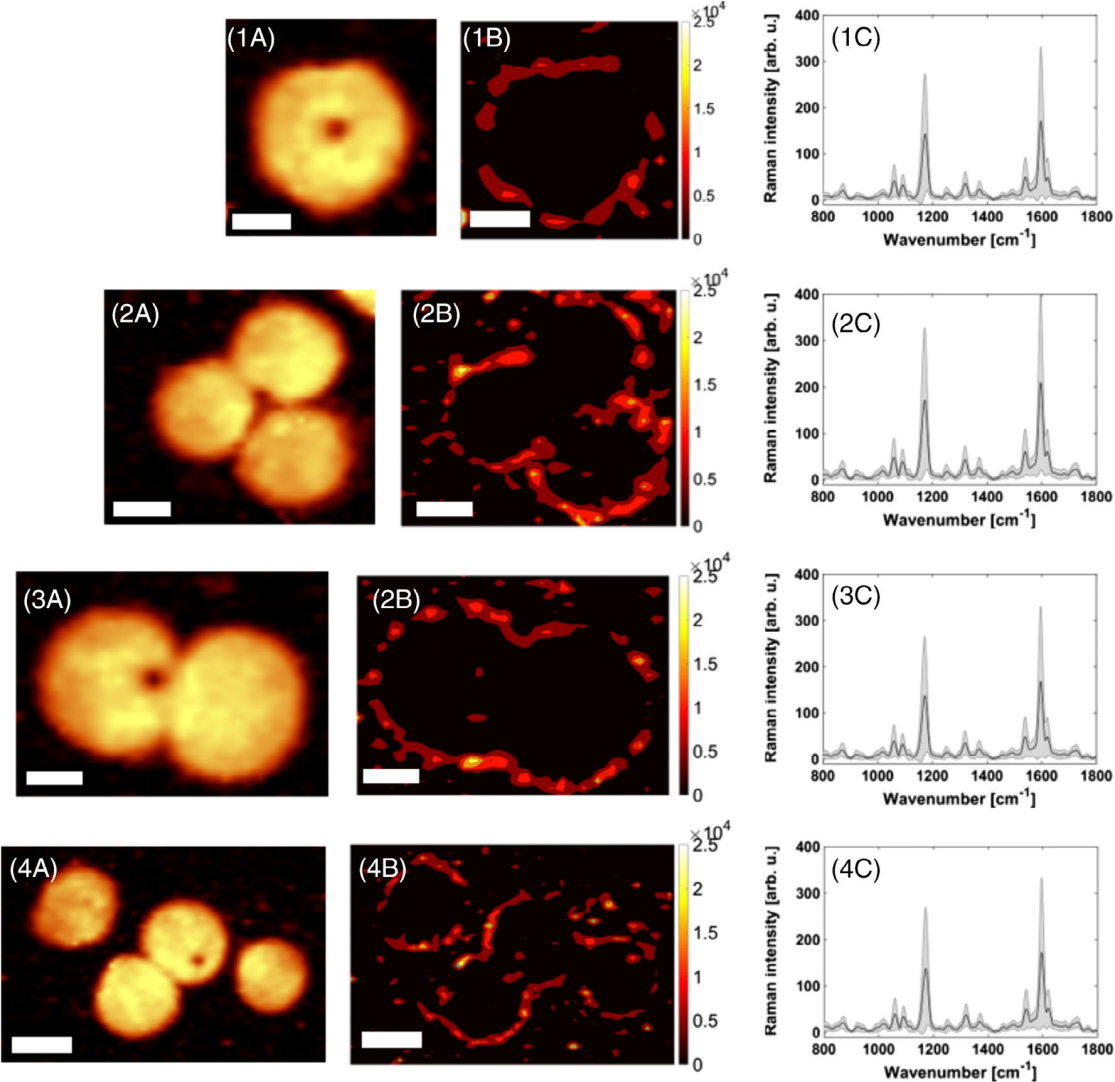
Localization of PD‐L1 on SkBr‐3 cells by iSERS microscopy. Fluorescence images (1A, 2A, 3A, 4A), iSERS false‐color images (1B, 2B, 3B, 4B) and mean SERS spectrum (1C, 2B, 3B, 4B). Scale bar is 5 μm. iSERS, immuno‐SERS microscopy; SERS, surface‐enhanced Raman scattering

**Figure 3:**
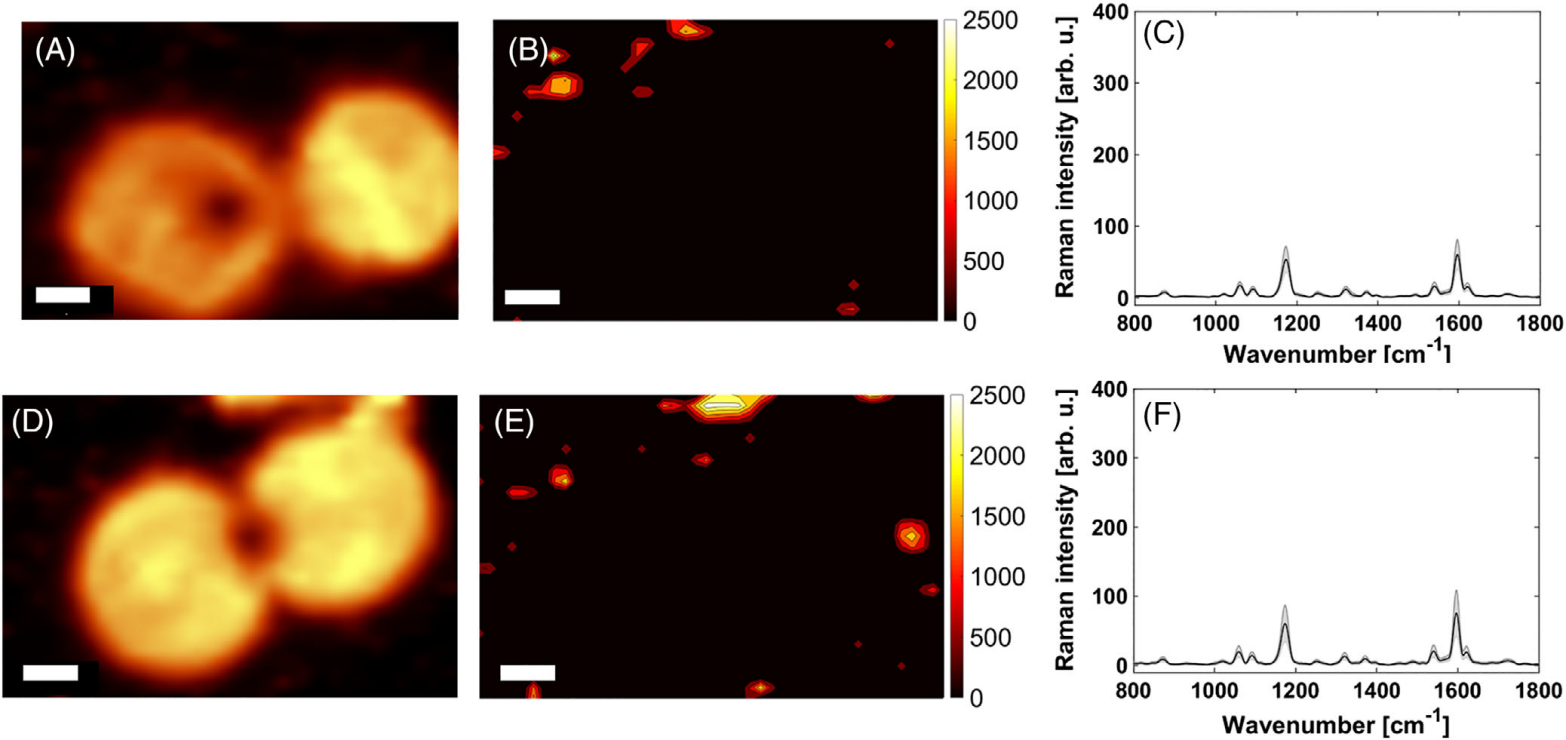
Visualization of the contributing low‐intensity pixels. A, D, Fluorescence images; B, E, false‐color images with reduced color scale bar; and C, F, mean SERS spectrum. Scale bar is 5 μm. SERS, surface‐enhanced Raman scattering

**Figure 4:**
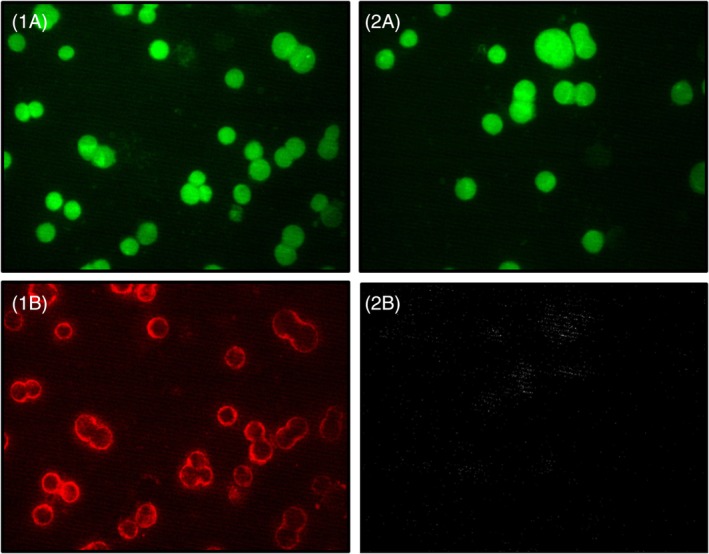
Four fluorescence images from two different samples (left column: sample 1 and right column: sample 2) with a green filter (1A and 2A) and with a red filter (1B and 2B). Sample 1 (left column) was incubated with both primary AB and the AF647‐labeled secondary AB. Sample 2 (right column) is a negative control and was incubated only with the AF647‐labeled secondary AB, but not with primary AB. The comparison of image 1B and 2B demonstrates specific binding of the primary anti‐PD‐L1 AB. The comparison of image 1A and 2A shows the autofluorescence upon excitation with a Hg lamp in combination with a 488 nm excitation filter. AB, antibody

Figure 2 shows the SERS mapping results for four single cells. The exact position of the cell is showed by the fluorescence visualization on the left side (Figures 1A, 2A, 3A and 4A). In the middle the scaled false‐color images are shown (Figures 1B, 2B, 3B and 4B), on the right side the corresponding mean SERS spectra with error bars showing the signal fluctuations (Figures 1C, 2C, 3C and 4C). Yellow and red pixels in the false‐color images indicate that the signals of the SERS nanotags were detected. For the interpretation of the false‐color SERS images only pixels with integrated Raman intensities larger than 100 counts (threshold manually set by user) were considered and the color scale was adjusted accordingly. The pattern of the false‐color SERS images shows that PD‐L1 can be selectively localized on the cell membrane of the SkBr‐3 cells.

Negative control experiments were performed in order to confirm the specificity of the staining. Specifically, SERS‐labeled secondary antibodies were incubated on SkBr‐3 cells without the previous addition of the primary antibody. Positive and negative controls were carried out under the same conditions. The corresponding false‐color images are depicted with the same color scale bar. The negative control (Figure S1A,C) shows no pixels with SERS signals above the threshold of 100 counts for the integrated Raman intensity. This suggests that no non–specific binding occurs at all. However, this is certainly not the case. The inspection of the corresponding mean SERS spectrum clearly reveals some detectable signals (Figure S1B,D).

The selective visualization of the contributing low‐intensity pixels requires an adjustment of the color scale bar; upon reduction of the upper limit from 25 000 counts (Figure S1A,C) to 2500 counts (Figure 3B,E) the low‐intensity pixels attributed to nonspecific binding become visible. In order to assess whether this non‐specific binding occur on the cells or on the glass slide, a visualization of the cells is required. We employed the integrated intensity from 300 to 2700 cm^−1^ which covers both SERS and especially also the underlying broad autofluorescence contribution from cellular chromophores. The corresponding image is depicted in Figure 3A,D. A comparison with Figure 3B,E suggests that non‐specific binding occurs dominantly on the glass substrate as the low‐intensity SERS pixels are mostly observed at positions where no cells are present.

In addition to iSERS experiments also immunofluorescence (IF) staining of the SkBr‐3 cells was performed. IF staining of PD‐L1 34 was carried out parallel to the iSERS staining experiments under the same conditions. The only difference is the incubation time with the fluorophore‐labeled secondary antibody (40 minutes for IF vs 8 hours for iSERS). We suppose that the reason for the longer incubation time for iSERS might be the overall size and weight of the SERS nanotags, causing slower binding to the primary antibodies. The fluorescence images are shown in Figure 4.

Overall, the presented false‐color images in Figure 2 demonstrate the capability of iSERS for selectively localizing PD‐L1 on single SkBr‐3 cells. The presented results confirm earlier iSERS PD‐L1 results on MDA‐MB‐231 cells 16.

Stable and bright molecularly functionalized gold nanoparticles (SERS nanotags) with a localized surface plasmon resonance (LSPR) in the red to near‐infrared are a pre‐requisite for obtaining high quality background‐free staining of target proteins such as PD‐L1 on colored and autofluorescent specimen. These optical considerations for background‐free imaging guide the rational design of SERS nanotags for iSERS microscopy on cells and tissue specimen. Au/Au core/satellite particles fulfill all necessary criteria: (a) they exhibit a LSPR in the red region of the UV/Vis extinction spectrum; (b) they are bright since plasmonic coupling between the Au core and the Au satellites leads to hot spots (very high local electric field strengths), which generate very strong SERS signals; (c) they are stable due to surface chemistry preventing the aggregation of the colloid 33. We employ Au/Au core/satellite particles with Raman‐active reporter molecules on the surface of the core 28. The unique Raman spectrum of the reporter molecule is enhanced by the gold nanostructure upon resonant optical excitation and is used for the identification of the SERS nanotag 19. In this proof of concept study we demonstrate the suitability of anti‐PD‐L1 antibody‐Au/Au/ core/satellite nanotag conjugates in iSERS microscopy for the selective localization of the predictive PD‐L1 marker on single SkBr‐3 cancer cells using SERS nanotags in combination with red laser excitation for minimizing interferences from background and autofluorescence. The negative control experiment with the omission of the primary anti‐PD‐L1 antibody confirms the binding specificity of the SERS‐labeled antibodies. Overall, these promising results on single cells pave the way for future high‐quality background‐free staining of PD‐L1 on tissue specimen. Future work will aim at the localization of PD‐L1 on FFPE tissue 35.

## Supporting information


**Appendix S1:** Supporting information

## References

[1] M. E. Keir , M. J. Butte , G. J. Freeman , A. H. Sharpe , Annu. Rev. Immunol. 2008, 26, 677. 18173375 10.1146/annurev.immunol.26.021607.090331 PMC10637733

[2] S. Ostrand‐Rosenberg , L. A. Horn , S. T. Haile , J. Immunol. 2014, 193, 8. 10.4049/jimmunol.1401572 PMC4185425 25281753

[3] H. Dong , S. E. Strome , D. R. Salomao , H. Tamura , F. Hirano , D. B. Flies , P. C. Roche , J. Lu , G. Zhu , K. Tamada , V. A. Lennon , E. Celis , L. Chen , Nat. Med. 2002, 8, 8. 10.1038/nm730 12091876

[4] H. Ghebeh , S. Mohammed , A. Al‐Omair , A. Qattan , C. Lehe , G. Al‐Qudaihi , N. Elkum , M. Alshabanah , S. Bin Amer , A. Tulbah , D. Ajarim , T. Al‐Tweigeri , S. Dermime , Neoplasia 2006, 8, 3. 10.1593/neo.05733 PMC1578520 16611412

[5] S. L. Topalian , C. G. Drake , D. M. Pardoll , Cancer cell 2015, 27, 450. 10.1016/j.ccell.2015.03.001 PMC4400238 25858804

[6] F. S. Hodi , S. J. O'Day , D. F. McDermott , R. W. Weber , J. A. Sosman , J. B. Haanen , R. Gonzalez , C. Robert , D. Schadendorf , J. C. Hassel , W. Akerley , A. J. van den Eertwegh , J. Lutzky , P. Lorigan , J. M. Vaubel , G. P. Linette , D. Hogg , C. H. Ottensmeier , C. Lebbe , C. Peschel , I. Quirt , J. I. Clark , J. D. Wolchok , J. S. Weber , J. Tian , M. J. Yellin , G. M. Nichol , A. Hoos , W. J. Urba , N. Eng. J. Med. 2010, 363, 13.

[7] J. Brahmer , K. L. Reckamp , P. Baas , L. Crino , W. E. Eberhardt , E. Poddubskaya , S. Antonia , A. Pluzanski , E. E. Vokes , E. Holgado , D. Waterhouse , N. Ready , J. Gainor , O. Aren Frontera , L. Havel , M. Steins , M. C. Garassino , J. G. Aerts , M. Domine , L. Paz‐Ares , M. Reck , C. Baudelet , C. T. Harbison , B. Lestini , D. R. Spigel , N. Eng. J. Med. 2015, 373, 2. 10.1056/NEJMoa1504627 PMC4681400 26028407

[8] A. M. Eggermont , V. Chiarion‐Sileni , J. J. Grob , R. Dummer , J. D. Wolchok , H. Schmidt , O. Hamid , C. Robert , P. A. Ascierto , J. M. Richards , C. Lebbe , V. Ferraresi , M. Smylie , J. S. Weber , M. Maio , C. Konto , A. Hoos , V. de Pril , R. K. Gurunath , G. de Schaetzen , S. Suciu , A. Testori , Lancet Oncol. 2015, 16, 5. 25840693 10.1016/S1470-2045(15)70122-1

[9] J. Larkin , V. Chiarion‐Sileni , R. Gonzalez , J. J. Grob , C. L. Cowey , C. D. Lao , D. Schadendorf , R. Dummer , M. Smylie , P. Rutkowski , P. F. Ferrucci , A. Hill , J. Wagstaff , M. S. Carlino , J. B. Haanen , M. Maio , I. Marquez‐Rodas , G. A. McArthur , P. A. Ascierto , G. V. Long , M. K. Callahan , M. A. Postow , K. Grossmann , M. Sznol , B. Dreno , L. Bastholt , A. Yang , L. M. Rollin , C. Horak , F. S. Hodi , J. D. Wolchok , N. Eng. J. Med. 2015, 373, 1. 10.1056/NEJMoa1504030 PMC5698905 26027431

[10] B. Weide , A. Martens , J. C. Hassel , C. Berking , M. A. Postow , K. Bisschop , E. Simeone , J. Mangana , B. Schilling , A. M. Di Giacomo , N. Brenner , K. Kahler , L. Heinzerling , R. Gutzmer , A. Bender , C. Gebhardt , E. Romano , F. Meier , P. Martus , M. Maio , C. Blank , D. Schadendorf , R. Dummer , P. A. Ascierto , G. Hospers , C. Garbe , J. D. Wolchok , Clin. Cancer Res. 2016, 22, 22. 10.1158/1078-0432.CCR-16-0127 PMC5572569 27185375

[11] P. C. Tumeh , C. L. Harview , J. H. Yearley , I. P. Shintaku , E. J. Taylor , L. Robert , B. Chmielowski , M. Spasic , G. Henry , V. Ciobanu , A. N. West , M. Carmona , C. Kivork , E. Seja , G. Cherry , A. J. Gutierrez , T. R. Grogan , C. Mateus , G. Tomasic , J. A. Glaspy , R. O. Emerson , H. Robins , R. H. Pierce , D. A. Elashoff , C. Robert , A. Ribas , Nature 2014, 515, 7528. 10.1038/nature13954 PMC4246418 25428505

[12] E. M. Van Allen , D. Miao , B. Schilling , S. A. Shukla , C. Blank , L. Zimmer , A. Sucker , U. Hillen , M. H. Foppen , S. M. Goldinger , J. Utikal , J. C. Hassel , B. Weide , K. C. Kaehler , C. Loquai , P. Mohr , R. Gutzmer , R. Dummer , S. Gabriel , C. J. Wu , D. Schadendorf , L. A. Garraway , Science 2015, 350, 6283. 10.1126/science.aad0095 PMC5054517 26359337

[13] L. Carbognin , S. Pilotto , M. Milella , V. Vaccaro , M. Brunelli , A. Calio , F. Cuppone , I. Sperduti , D. Giannarelli , M. Chilosi , V. Bronte , A. Scarpa , E. Bria , G. Tortora , PLoS One 2015, 10, 6. 10.1371/journal.pone.0130142 PMC4472786 26086854

[14] P. Schmid , S. Adams , H. S. Rugo , A. Schneeweiss , C. H. Barrios , H. Iwata , V. Dieras , R. Hegg , S. A. Im , G. Shaw Wright , V. Henschel , L. Molinero , S. Y. Chui , R. Funke , A. Husain , E. P. Winer , S. Loi , L. A. Emens , N. Eng. J. Med. 2018, 379, 22. 10.1056/NEJMoa1809615 30345906

[15] D. Schadendorf , D. E. Fisher , C. Garbe , J. E. Gershenwald , J. J. Grob , A. Halpern , M. Herlyn , M. A. Marchetti , G. McArthur , A. Ribas , A. Roesch , A. Hauschild , Nat. Rev. Dis. Primers 2015, 1, 15003. 27188223 10.1038/nrdp.2015.3

[16] J. A. Webb , Y.‐C. Ou , S. Faley , E. P. Paul , J. P. Hittinger , C. C. Cutright , E. C. Lin , L. M. Bellan , R. Bardhan , ACS Omega 2017, 2, 3583. 28782050 10.1021/acsomega.7b00527 PMC5537693

[17] S. Schlücker , Angew. Chem. Int. Edit. 2014, 53, 19. 10.1002/anie.201205748 24711218

[18] S. Schlücker , ChemPhysChem 2009, 10, 9. 10.1002/cphc.200900119 19565576

[19] S. Schlücker , B. Küstner , A. Punge , R. Bonfig , A. Marx , P. Ströbel , J. Raman Spectrosc. 2006, 37, 7.

[20] M. Schütz , S. Schlücker , PCCP 2015, 17, 37. 10.1039/c5cp03189c 26329892

[21] Y. Wang , S. Schlücker , Analyst 2013, 138, 8. 10.1039/c3an36866a 23420174

[22] M. Salehi , D. Steinigeweg , P. Ströbel , A. Marx , J. Packeisen , S. Schlücker , J. Biophotonics 2013, 6, 10. 10.1002/jbio.201200148 23225645

[23] X.‐P. Wang , Y. Zhang , M. König , E. Papadopoulou , B. Walkenfort , S. Kasimir‐Bauer , A. Bankfalvi , S. Schlücker , Analyst 2016, 141, 17. 10.1039/c6an00927a 27302205

[24] X.‐P. Wang , B. Walkenfort , M. König , L. König , S. Kasimir‐Bauer , S. Schlücker , Faraday Discuss. 2017, 205, 377. 10.1039/c7fd00135e 28902197

[25] Y. Zhang , S. Schlücker , in Confocal Raman Microscopy, Vol. 2 (Eds: Toporski J., Dieing T., Hollricher O.), Springer International Publishing, Basel, Switzerland 2018.

[26] J. H. Yoon , F. Selbach , L. Langolf , S. Schlücker , Small 2018, 14, 4. 10.1002/smll.201702754 29178555

[27] Q. Ruan , L. Shao , Y. Shu , J. Wang , H. Wu , Adv. Opt. Mater. 2014, 2, 1.

[28] V. Tran , B. Walkenfort , M. König , M. Salehi , S. Schlücker , Angew. Chem. Int. Edit. 2019, 58, 2. 10.1002/anie.201810917 PMC6582447 30288886

[29] P. H. C. Eilers , H. F. M. Boelens , Baseline Correction with Asymmetric Least Squares Smoothing, https://zanran_storage.s3.amazonaws.com/http://www.science.uva.nl/ContentPages/443199618.pdf (accessed: June 2019).

[30] J. H. Yoon , J. Lim , S. Yoon , ACS Nano 2012, 6, 8. 22827455 10.1021/nn302264f

[31] N. J. Halas , S. Lal , W.‐S. Chang , S. Link , P. Nordlander , Chem. Rev. 2011, 111, 6. 10.1021/cr200061k 21542636

[32] J. Wissler , M. Wehmeyer , S. Bäcker , S. Knauer , S. Schlücker , Anal. Chem. 2018, 90, 1. 29110458 10.1021/acs.analchem.7b02528

[33] V. Tran , C. Thiel , J. T. Svejda , M. Jalali , B. Walkenfort , D. Erni , S. Schlücker , Nanoscale 2018, 10, 46. 10.1039/c8nr06028b 30431039

[34] I. Grenga , R. N. Donahue , L. Lepone , J. Bame , J. Schlom , B. Farsaci , J. Immunother. Cancer 2014, 2(Suppl 3), P102.

[35] Y. Zhang , X.‐P. Wang , S. Perner , A. Bankfalvi , S. Schlücker , Anal. Chem. 2018, 90, 1. 29148719 10.1021/acs.analchem.7b03144

