# Supplementary material for: Localization of PD‐L1 on single cancer cells by iSERS microscopy with Au/Au core/satellite nanoparticles
Source: J Biophotonics. 2020 Jan 1;13(3):e201960034. doi: 10.1002/jbio.201960034 (PMC7065613; doi:10.1002/jbio.201960034)
Supplement: Supplementary file 1 — Appendix S1: Supporting information [file JBIO-13-e201960034-s001.pdf]

Supporting Information

Localization of PD-L1 on single cancer cells by iSERS microscopy with Au/Au core/satellite nanoparticles.

Elzbieta Stepula [1], Matthias König [1], Xin-Ping Wang [1], Janina Levermann [2], Tobias Schimming [3], Sabine Kasimir-Bauer [2], Bastian Schilling [3,4] and Sebastian Schlücker [1]*

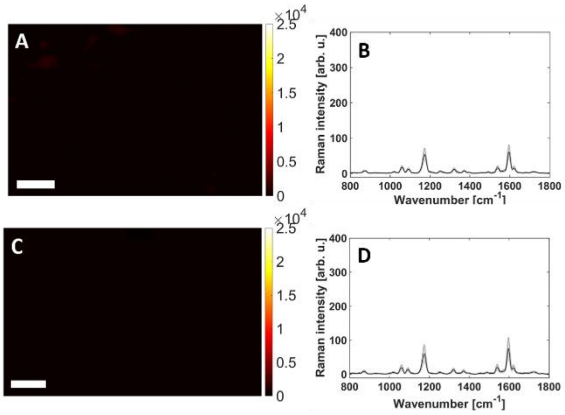

Figure S1. Negative control experiments. A and C: iSERS false-color images, B and D: mean value SERS spectrum. Scale bar is 5 µm.
